# Effect of Aspirin Challenge on Innate Lymphoid Cells in Asthma Patients With Aspirin Hypersensitivity

**DOI:** 10.1002/eji.70020

**Published:** 2025-08-04

**Authors:** Radosław Kacorzyk, Bogdan Jakiela, Alicja Maciejska, Agnieszka S. Węgrzyn, Adam Ćmiel, Marek Sanak, Lucyna Mastalerz

**Affiliations:** ^1^ 2nd Department of Internal Medicine Jagiellonian University Medical College Kraków Poland; ^2^ Doctoral School of Medical and Health Sciences Jagiellonian University Medical College Kraków Poland; ^3^ Department of Toxicological Biochemistry Faculty of Pharmacy Jagiellonian University Medical College Kraków Poland; ^4^ Łukasiewicz Research Network ‐ PORT Polish Center for Technology Development Bioengineering Group Wroclaw Poland; ^5^ Department of Applied Mathematics AGH University of Science and Technology Kraków Poland

**Keywords:** aspirin‐exacerbated respiratory disease, group 2 innate lymphoid cells, induced sputum, oral aspirin challenge

## Abstract

Previous studies confirmed increased group 2 innate lymphoid cell (ILC2s) count in nasal scrapings, alongside a reduced blood ILC2 count, in patients with nonsteroidal anti‐inflammatory drug‐exacerbated respiratory disease (N‐ERD) after intranasal administration of cyclooxygenase‐1 inhibitors. This study aimed to assess the role of blood and sputum ILCs in N‐ERD patients during oral aspirin‐induced bronchospasm and to compare patients with eosinophilic and noneosinophilic airway inflammatory phenotypes of asthma. Induced sputum, blood, and urine samples were collected in 24 patients with confirmed N‐ERD at baseline and during aspirin‐induced bronchospasm. Sputum and blood ILC counts were evaluated using flow cytometry. There was a significant increase in blood ILC1 count (*p *< 0.001) and percentage (*p* = 0.003) during aspirin‐induced bronchospasm. No significant changes in sputum ILCs were observed, but the number of detected ILCs was very low. There was a significant reduction in induced sputum supernatant (ISS) levels of prostaglandins PGE_2_ (*p* = 0.004) and PGD_2_ (*p* = 0.045), leukotriene B_4_ (*p* = 0.045), and 15‐oxo‐eicosatetraenoic acid (*p* = 0.045) during aspirin‐induced bronchospasm. Blood ILC1 count is increased during oral aspirin‐induced bronchospasm in patients with N‐ERD, but sputum ILC count at baseline is very low. Therefore, no reliable changes in sputum ILC counts can be detected during bronchospasm.

## Graphical abstract

In N‐ERD patients, oral aspirin‐induced bronchospasm is associated with increased blood group 1 innate lymphoid cells (ILC1s), decreased sputum eicosanoids, and increased urinary leukotriene E4 (LTE4), suggesting a potential role of blood ILC1s in aspirin‐induced bronchoconstriction.

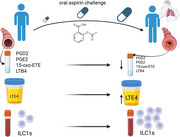

**Abbreviations**
12‐HETE: 12‐hydroxyeicosatetraenoic acid
15‐HETE: 15‐hydroxyeicosatetraenoic acid
15‐oxo‐ETE: 15‐oxo‐eicosatetraenoic acid
COX‐1: cyclooxygenase type 1
CRTH2: chemoattractant receptor‐homologous molecule expressed on Th2 cells
HPLC‐MS/MS: high‐performance liquid chromatography‐tandem mass spectrometry
ILC1s: group 1 innate lymphoid cells
ILC2s: group 2 innate lymphoid cells
ILC3s: group 3 innate lymphoid cells
ILCs: innate lymphoid cells
ISS: induced sputum supernatant
LTB_4_
: leukotriene B_4_
LTE_4_
: leukotriene E_4_
N‐ERD: nonsteroidal anti‐inflammatory drug‐exacerbated respiratory disease
PGD_2_
: prostaglandin D_2_
PGE_2_
: prostaglandin E_2_
TSLP: thymic stromal lymphopoietin

## 1 Introduction

Nonsteroidal anti‐inflammatory drug‐exacerbated respiratory disease (N‐ERD) is characterized by a triad of asthma, chronic rhinosinusitis with nasal polyps (CRSwNP), and respiratory symptoms after ingestion of cyclooxygenase type 1 (COX‐1) inhibitors [1]. N‐ERD is marked by overproduction of cysteinyl leukotrienes, prostaglandin D_2_ (PGD_2_), and alarmins: interleukin (IL)‐25, IL‐33, and thymic stromal lymphopoietin (TSLP) [2, 3, 4, 5]. These mediators play a key role in type 2 (T2) inflammation in N‐ERD and can activate group 2 innate lymphoid cells (ILC2s) [2, 6, 7, 8, 9].

Innate lymphoid cells (ILCs) are lineage‐negative cells of innate immunity, characterized by the expression of the interleukin 7 receptor α (IL‐7Rα, CD127) [10]. ILCs are categorized into three subtypes: group 1 ILCs (ILC1s), ILC2s, and group 3 ILCs (ILC3s), corresponding to specific CD4+ T helper (T_h_) cells of adaptive immunity: T_h_1, T_h_2, and T_h_17/22 cells, respectively [10].

ILC2s express the chemoattractant receptor‐homologous molecule expressed on Th2 (CRTH2, CD294), variable levels of c‐Kit (CD117), and the transcription factor GATA‐binding protein 3 (encoded by *GATA3*) [10, 11, 12]. Additionally, ILC2s produce cytokines characteristic of T2 inflammation: IL‐4, IL‐5, and IL‐13, contributing to tissue eosinophilia and anti‐helminth immunity [13, 14, 15, 16, 17]. In contrast, ILC1s do not express CRTH2 or CD117 but express T‐box‐expressed‐in‐T cells (encoded by *TBX21*) [13]. They also produce interferon‐γ and are involved mostly in antiviral immunity [13]. ILC3s express CD117 but not CRTH2 [13]. They also express the retinoic acid receptor‐related orphan receptor‐γt and produce cytokines such as IL‐17A and IL‐22. ILC3s are primarily involved in anti‐bacterial immunity [13] and neutrophilic inflammatory phenotype [18]. Trans‐differentiation between ILC groups has already been described [19, 20, 21].

ILCs are tissue‐resident cells [22, 23] that are particularly abundant at mucosal barriers such as the bronchial and nasal mucosa [13, 24]. However, upon activation, ILC2s may leave the bone marrow and be recruited to sites of inflammation [25, 26].

In patients with NERD, aspirin‐induced inhibition of COX1 causes an imbalance in eicosanoid synthesis, reducing anti‐inflammatory prostaglandin E_2_ (PGE_2_) levels and increasing the production of cysteinyl leukotrienes and PGD_2_ [2, 3, 27]. These lipid mediators subsequently bind to their receptors on ILC2s: cysteinyl leukotriene receptor 1 (CysLT1R) and CRTH2, respectively, causing ILC2 activation [2, 3, 6, 7, 27]. Data on the effect of aspirin on ILC1s and ILC3s are lacking.

So far, the role of ILC2s in N‐ERD has been studied only in the upper airways [28, 29]. Previous studies confirmed an increase in ILC2 count in nasal scrapings, alongside a decrease in blood ILC2s in patients with N‐ERD after intranasal administration of a COX‐1 inhibitor [28]. One potential mechanism for ILC2 recruitment to the nasal mucosa may be the interaction of PGD_2_ with the CRTH2 receptor expressed on ILC2s [7, 11, 30]. Recent research indicated that baseline serum levels of 15‐hydroxyeicosatetraenoic acid (15‐HETE) inversely correlate with an increase in ILC2 count in nasal scrapings, while serum levels of 19,20‐dihydroxy‐4Z,7Z,10Z,13Z,16Z‐docosapentaenoic acid positively correlate with nasal ILC2 accumulation in N‐ERD patients after intranasal COX‐1 inhibitor challenge [29].

To date, sputum ILCs have been described in the general asthma population [31, 32, 33, 34]. It was shown that ILC2 count was increased in the sputum of adult asthma patients 24 h after allergen challenge, while blood ILC2 count was reduced, suggesting the trafficking of circulating ILC2s to the lungs [33]. Kim et al. [34] reported elevated ILC1, ILC2, and ILC3 counts in induced sputum from treatment‐naïve patients with asthma compared with healthy controls. Notably, sputum ILC2 count was significantly higher in patients with eosinophilic asthma compared with those with noneosinophilic asthma [34].

To our knowledge, sputum ILCs have not been studied in N‐ERD. We hypothesized that the activation and recruitment of ILCs, particularly ILC2s, may play a role in the mechanisms underlying acute reactions to aspirin and inflammation in N‐ERD. This study aimed to assess the role of ILCs in sputum and blood in patients with N‐ERD at baseline (prior to oral aspirin challenge) and during acute bronchospasm induced by aspirin. Another objective was to assess differences between patients with eosinophilic and noneosinophilic asthma phenotypes. To achieve this, a clinical study was conducted in which patients underwent a 4‐day hospitalization for an oral aspirin challenge.

## 2 Results

### 2.1 Effect of Oral Aspirin Challenge on Blood and Sputum Parameters in all Study Patients

**FIGURE 1:**
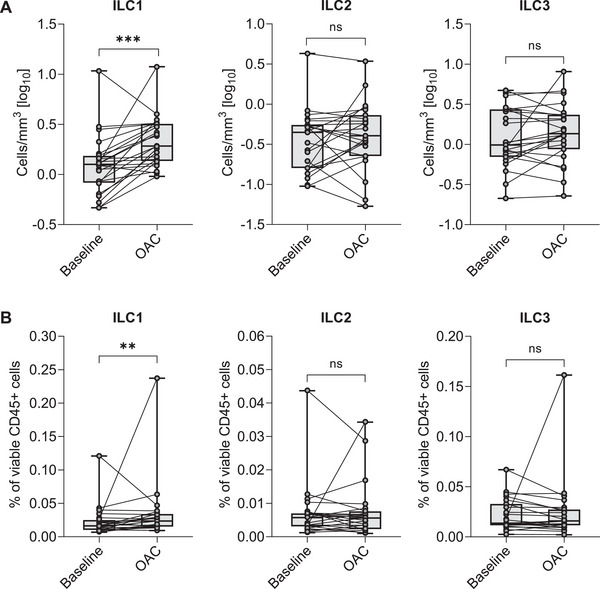
Effect of oral aspirin challenge on blood innate lymphoid cells in patients with N‐ERD: (A) Innate lymphoid cells count. (B) Innate lymphoid cells percentage. Data are presented as medians with 0.25 and 0.75 quartiles (*n* = 24). Wilcoxon test: ***p < 0.001, **p < 0.01, ns: not significant. ILC1, group 1 innate lymphoid cells; ILC2, group 2 innate lymphoid cells; ILC3s, group 3 innate lymphoid cells; OAC, oral aspirin challenge.

There was a significant increase in blood ILC1 count (1.261 cells/mm^3^ at baseline vs. 1.911 cells/mm^3^ postchallenge; *p* < 0.001) and percentage (0.016% vs. 0.023%; *p* = 0.003) during aspirin‐induced bronchospasm compared with baseline (see Figure 1). No significant changes in ILC2 and ILC3 counts and percentages were observed. Additionally, no significant changes were observed in sputum cell percentages and inflammatory sputum phenotypes during aspirin‐induced bronchospasm compared with baseline. Moreover, no significant changes in sputum ILCs were observed. However, data regarding sputum ILCs are less reliable due to the very low ILC counts detected in sputum samples. No changes were observed in the relative expression of the evaluated gene mRNA markers. There were no changes in blood and ISS cytokine levels, including alarmins and T2 cytokines. Data are presented in Table 1 and Table E1 in the Supporting Information.

**TABLE 1:** Blood and sputum parameters at baseline and during aspirin‐induced bronchospasm in the N‐ERD cohort (*n* = 24).

Variable	At baseline	During aspirin‐induced bronchospasm	BH adjusted *p*‐value
Blood cell counts [cells × 10^3^/mm^3^]			
**WBC**	**7.79 [6.14; 8.61]**	**9.36 [7.11; 10.71]**	**0.008**
**Neutrophils**	**4.09 [3.15; 5.55]**	**6.32 [4.17; 7.07]**	**0.008**
**Eosinophils**	**0.36 [0.27; 0.71]**	**0.31 [0.11; 0.48]**	**0.041**
Lymphocytes	1.88 [1.68; 2.30]	2.01 [1.74; 2.58]	0.130
Monocytes	0.56 [0.41; 0.70]	0.6 [0.37; 0.77]	0.831
Basophils	0.05 [0.04; 0.08]	0.06 [0.02; 0.08]	0.554
Blood cell counts [%]			
Neutrophils	59.25 [51.63; 64.53]	62 [56.95; 71.85]	0.156
**Eosinophils**	**5.7 [3.28; 8.28]**	**3.5 [1.40; 6.65]**	**0.011**
Lymphocytes	25.45 [21.80; 31.00]	24.1 [19.50; 27.95]	0.368
**Monocytes**	**7.4 [6.55; 8.60]**	**6.9 [5.95; 7.60]**	**0.008**
**Basophils**	**0.75 [0.60; 0.90]**	**0.6 [0.40; 0.75]**	**0.015**
Blood ILC count [cells/mm^3^]			
**ILC1s**	**1.261 [0.849; 1.479]**	**1.911 [1.378; 3.067]**	**<0.001**
ILC2s	0.453 [0.160; 0.569]	0.404 [0.263; 0.692]	0.558
ILC3s	1.01 [0.745; 2.733]	1.36 [0.934; 2.304]	0.410
Blood ILC percentages [% of viable CD45+cells]			
**ILC1s**	**0.016 [0.011; 0.023]**	**0.023 [0.015; 0.031]**	**0.003**
ILC2s	0.006 [0.003; 0.007]	0.006 [0.003; 0.007]	0.808
ILC3s	0.015 [0.012; 0.034]	0.016 [0.012; 0.027]	0.403
Induced sputum ILCs [% of viable CD45+ sputum cells]			
ILC1s	0.01 [0.01; 0.04]	0.01 [0.01; 0.01]	0.388
ILC2s	0.01 [0.01; 0.01]	0.01 [0.01; 0.01]	0.180
ILC3s	0.01 [0.01; 0.03]	0.01 [0.01; 0.08]	0.501
ISS eicosanoid concentration [pg/mg protein]			
**Leukotriene B4**	**285.17 [162.78; 362.50]**	**91.99 [63.72; 234.30]**	**0.045**
Leukotriene C4	0.44 [0.44; 4.42]	0.72 [0.44; 7.78]	0.395
Leukotriene D4	13.89 [6.55; 18.58]	17.64 [8.28; 25.18]	0.575
Leukotriene E4	61.64 [11.18; 154.17]	123.54 [26.35; 257.03]	0.337
**Prostaglandin D2**	**22.67 [15.46; 35.70]**	**13.39 [7.01; 23.59]**	**0.045**
**Prostaglandin E2**	**136.87 [57.98; 197.88]**	**54.58 [25.13; 97.62]**	**0.004**
5‐HETE	529.09 [294.49; 873.69]	231.73 [118.52; 464.76]	0.054
12‐HETE	1298.22 [700.89; 2505.84]	1029.26 [422.8; 1599.7]	0.146
15‐HETE	1213.69 [465.44; 4291.70]	885.27 [284.02; 2402.33]	0.258
** 15‐oxo‐ETE**	**139.35 [118.11; 278.47]**	**52.87 [36.78; 111.17]**	**0.045**

*Note*: Values are expressed as medians [0.25; 0.75 quartiles]. Detection threshold for blood ILCs was ∼0.001% of viable CD45+ cells. Detection threshold for sputum ILCs was ∼0.01% of viable CD45+ sputum cells. A *p*‐value of less than 0.05 was considered statistically significant.

Abbreviations: BH, Benjamini‐Hochberg; ILCs, innate lymphoid cells; ISS, induced sputum supernatant; WBC, white blood cells; 5‐HETE, 5‐hydroxyeicosatetraenoic acid; 12‐HETE, 12‐hydroxyeicosatetraenoic acid; 15‐HETE, 15‐hydroxyeicosatetraenoic acid; 15‐oxo‐ETE, 15‐oxo‐eicosatetraenoic acid

### 2.2 Asthma Phenotypes in N‐ERD Patients at Baseline

Based on baseline sputum cell percentage, 10 patients (42%) had the eosinophilic asthma phenotype, 13 patients (54%) had the paucigranulocytic phenotype, and 1 patient (4%) had the neutrophilic phenotype. For further analyses, patients were divided into two groups: those with the eosinophilic asthma phenotype (10 patients, 42%) and those with the noneosinophilic asthma phenotype, which included the paucigranulocytic and neutrophilic phenotypes (14 patients, 58%). As expected, patients with the eosinophilic asthma phenotype had significantly increased IS‐cell expression of eosinophil signature mRNA markers: *CLC* (*p *< 0.001) and *PRSS33* (*p *< 0.004), as well as important T2 marker *CLCA1* (*p* = 0.002). However, no differences were observed in ISS cytokine levels. Additionally, no differences were found in clinical variables between the groups. Patients with the eosinophilic phenotype had a higher baseline blood eosinophil count (550 cells/mm^3^ vs. 300 cells/mm^3^, *p* = 0.022) compared with patients with the noneosinophilic phenotype. The characteristics of the groups are presented in Table 2.

**TABLE 2:** Baseline characteristics of the N‐ERD cohort depending on the asthma phenotype.

Variable	All patients (*n* = 24)	Patients with eosinophilic asthma phenotype (*n* = 10)	Patients with noneosinophilic asthma phenotype (*n* = 14)	BH‐adjusted *p*‐value^a^
Age (years)	46 [41.3; 54.5]	49 [39; 54]	45.5 [42; 56]	0.666
Women	20 (83.3)	7 (70)	13 (92.86)	0.272
BMI (kg/m^2^)	25.6 [23.1; 29.3]	25.6 [24.2; 27.4]	26.4 [22.4; 30.5]	0.931
Age of asthma onset (years)	33.5 [24; 40.8]	39 [26; 44]	31.5 [21; 37]	0.770
Asthma duration (years)	12 [9; 21]	14 [7; 20]	10.5 [9; 25]	0.709
Asthma severity				
Mild	0 (0)	0	0	
Moderate	14 (58.3)	5 (50)	9 (64.29)	0.678
Severe	10 (41.7)	5 (50)	5 (35.71)	
ICS dose (µg/day; fluticasone propionate or equivalent)	500 [400; 1000]	550 [400; 1500]	450 [400; 800]	0.259
Exacerbations in the past year	0 [0; 2]	0 [0; 2]	1 [0; 2]	0.643
PD20 of aspirin (mg)	350 [150; 420]	355 [102.5; 410]	350 [150; 430]	0.916
FEV1 (% predicted)	96 [86; 105]	95 [83; 106]	97 [90; 105]	1.0
ACT	23 [20; 25]	23 [21; 25]	22.5 [20; 25]	0.796
ACQ‐7	0.7 [0.4; 1.4]	0.72 [0.43; 1.3]	0.79 [0.43; 1.7]	0.796
AQLQ	5 [4.6; 6.2]	5.04 [4.73;5.73]	5.27 [4.2; 6.33]	0.931
CRSwNP	24 (100)	10 (100)	14 (100)	1.0
SNOT‐22	49 [36.8; 62.8]	49 [36; 71]	50.5 [37; 62]	0.796
Lund‐Mackay score	18 [13; 20]	18 [15; 20]	16 [9.5; 20.5]	0.508
Positive skin prick test	8 (33.3)	3 (30)	5 (36)	0.639
Serum IgE (IU/mL)	118 [74.4; 184]	101.75 [71.3; 324]	119.5 [81.2; 164]	0.796
**Blood eosinophil count (×10^3^/mm^3^)**	0.36 [0.27; 0.71]	**0.55 [0.38; 0.76]**	**0.3 [0.2; 0.37]**	**0.022**
**Blood eosinophil %**	5.7 [3.3; 8.3]	**7.9 [5.6; 9.0]**	**3.7 [2.5; 6.5]**	**0.026**

*Note*: Values are expressed as medians [0.25; 0.75 quartiles] or *n* (%). Asthma severity was assessed according to the 2022 GINA guidelines [48]. A *p*‐value of less than 0.05 was considered statistically significant.

Abbreviations: ACT, asthma control test; ACQ‐7, 7‐item asthma control questionnaire; AQLQ, asthma quality of life questionnaire; BH, Benjamini–Hochberg; BMI, body mass index; CRSwNP, chronic rhinosinusitis with nasal polyps; FEV_1_, forced expiratory volume in 1 second; ICS, inhaled corticosteroids; IgE, immunoglobulin E; PD20, provocative dose of aspirin causing a 20% drop in FEV_1_; SNOT‐22, sino‐nasal outcome test‐22

^a^ BH adjusted *p*‐value: N‐ERD patients with eosinophilic asthma phenotype (*n* = 10) versus N‐ERD patients with noneosinophilic asthma phenotype (*n* = 14).

### 2.3 Blood and Sputum ILCs in Patients with Different Asthma Phenotypes

No significant differences were found in blood ILC count and percentage at baseline between patients with eosinophilic and noneosinophilic asthma phenotypes. However, patients with the eosinophilic phenotype had a higher sputum ILC3 percentage at baseline compared with those with the noneosinophilic phenotype (0.026% vs. 0.01%, *p* = 0.048). No differences were found in sputum ILC1 and ILC2 percentages. Details are presented in Table 3.

**TABLE 3:** Differences between N‐ERD patients with eosinophilic (*n* = 10) and noneosinophilic asthma phenotype (*n* = 14) at baseline.

Variable	Patients with eosinophilic asthma phenotype (*n* = 10)	Patients with non‐eosinophilic asthma phenotype (*n* = 14)	BH‐adjusted *p*‐value
Blood ILC count [cells/mm^3^]			
ILC1s	1.11 [0.53; 1.45]	1.30 [0.86; 1.56]	0.285
ILC2s	0.39 [0.14; 0.53]	0.50 [0.20; 0.65]	0.259
ILC3s	0.98 [0.57; 2.1]	1.01 [0.83; 2.89]	0.437
Blood ILC percentages [% of viable CD45+cells]			
ILC1s	0.01 [0.01; 0.02]	0.02 [0.01; 0.03]	0.371
ILC2s	0.01 [0.00; 0.01]	0.01 [0.00; 0.01]	0.403
ILC3s	0.01 [0.01; 0.02]	0.02 [0.01; 0.04]	0.437
Induced sputum ILCs [% of viable CD45+ sputum cells]			
ILC1s	0.01 [0.01; 0.05]	0.01 [0.01; 0.01]	0.312
ILC2s	0.01 [0.01; 0.01]	0.01 [0.01; 0.01]	0.437
**ILC3s**	**0.03 [0.01; 0.07]**	**0.01 [0.01; 0.01]**	**0.048**
ISS eicosanoid concentration [pg/mg protein]			
**Leukotriene B_4_**	**164.34 [75.8; 177.71]**	**348.08 315.8; 423.7]**	**0.001**
Leukotriene C_4_	0.44 [0.44; 0.44]	1.95 [0.44; 5.35]	0.212
Leukotriene D_4_	18.16 [6.60’ 42.49]	9.55 [6.39; 16.94]	0.212
**Leukotriene E_4_**	**176.50 [85.08; 390.77]**	**25.86 [9.04; 70.48]**	**0.006**
Prostaglandin D_2_	28.97 [16.84; 58.74]	19.8 [15.08; 30.21]	0.212
Prostaglandin E_2_	143.16 [58.46; 177.08]	136.87 [56.53; 285.26]	0.625
**5‐HETE**	**336.87 [137.14; 539.35]**	**651.2 [428.09; 945.99]**	**0.031**
**12‐HETE**	**691.46 [546.91; 1302.28]**	**2108.11 [1274.97; 2939.18]**	**0.009**
**15‐HETE**	**4810.64 [1324.14; 6721.81]**	**639.38 [400.03; 1225.98]**	**0.009**
15‐oxo‐ETE	139.35 [131.30; 213.58]	147.58 [79.67; 362.24]	0.709

*Note*: Values are expressed as medians [with 0.25; 0.75 quartiles]. Detection threshold for blood ILCs was ∼0.001% of viable CD45+ cells. Detection threshold for sputum ILCs was ∼0.01% of viable CD45+ sputum cells. A *p*‐value of less than 0.05 was considered statistically significant.

Abbreviations: BH, Benjamini–Hochberg; ILCs, innate lymphoid cells; ISS, induced sputum supernatant; 5‐HETE, 5‐hydroxyeicosatetraenoic acid; 12‐HETE, 12‐hydroxyeicosatetraenoic acid; 15‐HETE, 15‐hydroxyeicosatetraenoic acid; 15‐oxo‐ETE, 15‐oxo‐eicosatetraenoic acid.

### 2.4 Effect of Oral Aspirin Challenge on Sputum and Blood Parameters in Patients with Different Asthma Phenotypes

A significant increase in blood ILC1 count was observed after the aspirin challenge in patients with noneosinophilic asthma (1.299 cells/mm^3^ at baseline vs. 1.929 cells/mm^3^ postchallenge; *p* = 0.001) and those with eosinophilic asthma (1.112 cells/mm^3^ vs. 1.863 cells/mm^3^; *p* = 0.009). Similarly, a significant increase in blood ILC1 percentage was noted (noneosinophilic asthma: 0.019% at baseline vs. 0.024% postchallenge; *p* = 0.013 and eosinophilic asthma: 0.014% vs. 0.021%; *p* = 0.049). However, no significant changes were found in blood ILC2 and ILC3 in either group. Additionally, no significant changes were observed in sputum ILCs during aspirin‐induced bronchospasm compared with baseline in either group. Finally, no changes were detected in blood and ISS cytokine concentrations during aspirin‐induced bronchospasm compared with baseline in any of the groups.

### 2.5 Lipid Mediators in ISS at Baseline and during Aspirin‐Induced Bronchospasm

**FIGURE 2:**
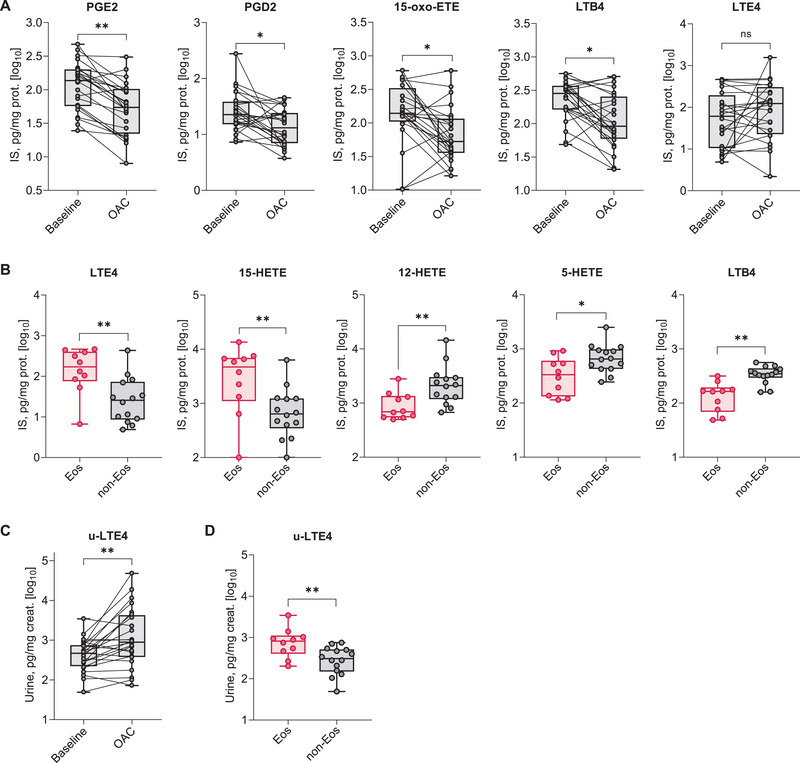
Eicosanoids in induced sputum supernatant (ISS) and urine. Effect of oral aspirin challenge on ISS (A) and urine (C) eicosanoids. Data presented as medians with 0.25 and 0.75 quartiles (*n* = 24). Wilcoxon test: **p < 0.01, *p < 0.05, ns: not significant. Differences in baseline ISS (B) and urine (D) eicosanoids between patients with eosinophilic (*n* = 10) and noneosinophilic (*n* = 14) asthma phenotypes. Data presented as medians with 0.25 and 0.75 quartiles. Mann–Whitney *U* test: **p < 0.01, *p < 0.05. 12‐HETE, 12‐ hydroxyeicosatetraenoic acid; 15‐HETE, 15‐hydroxyeicosatetraenoic acid; 15‐oxo‐ETE, 15‐oxo‐eicosatetraenoic acid; Eos, eosinophilic asthma phenotype; IS, induced sputum; LTB_4_, leukotriene B_4_; non‐eos, noneosinophilic asthma phenotype; OAC, oral aspirin challenge; PGD_2_, prostaglandin D_2_; PGE_2_, prostaglandin E_2_; u‐LTE_4_, urinary leukotriene E_4_.

There was a significant reduction in ISS levels of PGE_2_ (136.87 pg/mg protein vs. 54.58; *p* = 0.004), PGD_2_ (22.67 vs. 13.39; *p* = 0.045), LTB_4_ (285.17 vs. 91.99; *p* = 0.045), and 15‐oxo‐ETE (139.35 vs. 52.87; *p* = 0.045) during aspirin‐induced bronchospasm compared with baseline (see Figure 2A). No significant changes were observed in ISS levels of LTE_4_ (61.64 pg/mg protein vs. 123.54; *p* = 0.337) and other cysteinyl leukotrienes. The observed changes were independent of the inflammatory asthma phenotype. Details are presented in Table 1 and Table E1 in the Supporting Information.

At baseline, patients with the eosinophilic asthma phenotype had higher ISS levels of LTE_4_ (176.50 pg/mg protein vs. 25.86; *p* = 0.006) and 15‐HETE (4810.64 vs. 639.38; *p* = 0.009) and lower ISS levels of LTB_4_ (164.34 vs. 348.08; *p* = 0.001), 5‐HETE (336.87 vs. 651.2; *p* = 0.031), and 12‐HETE (691.45 vs. 2108.11; *p* = 0.009) compared with those with the noneosinophilic asthma phenotype (see Figure 2B). Details are presented in Table 3 and Table E2 in the Supporting Information.

### 2.6 Lipid Mediators in Urine at Baseline and During Aspirin‐Induced Bronchospasm

A significant increase in urinary LTE_4_ levels was observed during aspirin‐induced bronchospasm compared with baseline (466.82 pg/mg creatinine vs. 894.99; *p* = 0.009) (see Figure 2C). No significant changes in other urinary eicosanoids were observed. Similar to ISS, these changes were independent of the inflammatory asthma phenotype. Details are presented in Table E1 in the Supporting Information.

Patients with the eosinophilic asthma phenotype had increased urinary LTE_4_ levels (814.52 pg/mg creatinine vs. 308.29; *p* = 0.009) compared with those with the noneosinophilic asthma phenotype (see Figure 2D). No differences in other urinary eicosanoids were observed. Details are presented in Table E2 in the Supporting Information.

### 2.7 Correlations Between Lipid Mediators and Inflammatory Cells at Baseline

There was a significant positive correlation between urinary LTE_4_ and blood eosinophil count (*r* = 0.56, *p* = 0.004) and sputum eosinophil percentage (*r* = 0.51, *p* = 0.01). Moreover, ISS LTE_4_ levels correlated positively with sputum eosinophil percentage (*r* = 0.66, *p *< 0.001) and expression of eosinophil signature genes (e.g., *CLC r* = 0.74, *p *< 0.001) and T2 genes (e.g., *CLCA1 r* = 0.77, *p *< 0.001). No significant correlation was found between ISS and urinary LTE_4_ levels (*r* = 0.05, *p* = 0.828). Additionally, no significant correlations were found between blood ILCs and lipid mediators in ISS and urine. Due to the very low sputum ILC count, correlations between lipid mediators and sputum ILCs were not assessed.

## 3 Discussion

To our knowledge, this is the first study to assess changes in blood and sputum ILCs after aspirin‐induced bronchospasm in patients with N‐ERD. Our findings demonstrated that blood ILC1 count and percentage increased during aspirin‐induced bronchospasm compared with baseline. ILC1s produce interferon‐γ and are involved in antiviral immunity, similar to T_h_1 cells [13], and they are associated with the noneosinophilic inflammatory phenotype in asthma [35].

Although previous in vitro and murine studies have demonstrated that the conversion of ILC2s into ILC1s can occur over several days in the presence of IL‐2 and IL‐12 or IL‐1β and IL‐12 [20, 36], it remains unclear whether such a phenotypic shift can take place within hours in humans following an oral aspirin challenge. Given the rapid sampling in our study, the observed changes in ILCs subsets may reflect early activation or redistribution rather than skewed transdifferentiation, and further studies are needed to elucidate the kinetics and mechanisms of this process in vivo. No changes were observed in blood ILC2s and ILC3s.

Surprisingly, we found no significant changes in the percentage of sputum ILC1s, ILC2s, and ILC3s during aspirin‐induced bronchospasm. ILC2s were below the detection threshold at baseline and did not increase during aspirin‐induced bronchospasm in sputum. The low count of sputum ILCs and the lack of changes during aspirin‐induced bronchospasm may be due to ILCs primarily residing deep in the bronchial mucosa or submucosa and lung tissues [13, 19, 20, 21]. Another reason could be the limited availability of sputum ILCs [37], and the exact mechanisms of ILC migration and persistence within sputum are unknown. Bronchial mucosa sampling, bronchial scraping, or bronchoalveolar lavage (BAL) fluid might provide better material for evaluating ILCs. Importantly, Monticelli et al. [38] first identified ILCs in the human respiratory tract and lung parenchyma, detecting ILCs in BAL fluid and human lung tissue from cadaver organ donors [38]. The lack of an increase in sputum ILC percentage may be due to insufficient time between aspirin administration and sputum collection. It is possible that ILC2s in sputum should be assessed at a different time point, such as 24 h after aspirin challenge, similar to allergen challenge [33]. A significant increase in total ILC2s, as well as IL‐5^+^, IL‐13^+^, and CRTH2^+^ ILC2s, was observed in sputum 24 h after allergen exposure, coinciding with a significant decrease in blood ILC2s. Notably, airway eosinophilia correlated with IL‐5^+^ ILC2s at all time points after allergen exposure [33]. ILCs were analyzed immediately after the onset of aspirin‐induced bronchospasm, before administration of glucocorticosteroids and antihistamines, to avoid treatment‐related alterations in cell counts.

Eastman et al. described an increase in ILC2 count in nasal scrapings, accompanied by a decrease in blood ILC2 count in N‐ERD patients after intranasal administration of a COX‐1 inhibitor [28]. Differences in the migration of ILCs from blood to the upper and lower respiratory tracts during the reaction induced by the COX‐1 inhibitor may be attributed to the following factors: (1) the route of COX‐1 inhibitor administration (intranasal vs. oral); (2) the invasiveness of cell collection methods (nasal scraping vs. sputum); (3) the trauma associated with obtaining nasal scrapings and induced sputum samples could, in itself, cause adverse reactions in the upper and lower respiratory tracts.

The potential mechanism underlying the recruitment and activation of ILC2s in the bronchi may involve binding of PGD_2_ to the CRTH2 receptors on ILC2s [7, 27, 33]. During aspirin‐induced bronchospasm, we observed a decrease in both proinflammatory PGD_2_ and anti‐inflammatory PGE_2_ levels, with no changes in the urinary PGD_2_ metabolite. The reduction in ISS PGD_2_ levels could explain the absence of ILC2 recruitment to sputum. In our previous research, we found no increase in PGD_2_ in ISS, with levels remaining constant during acute reactions after aspirin challenge [39]. However, a consistent decrease in anti‐inflammatory PGE_2_ levels was noted [39, 40]. Cahill et al. [41] reported an increase in the urinary PGD_2_ metabolite during oral aspirin challenge in patients with aspirin hypersensitivity. In contrast, Bochenek et al. observed an increase in blood 9α,11β‐PGF_2_, a major stable PGD_2_ metabolite, during oral aspirin challenge, with no significant changes in urinary levels of the PGD_2_ metabolite [42]. Therefore, ISS PGD_2_ levels may not accurately represent changes in blood and urine. We also hypothesized that ILC2s could produce substantial amounts of T2 cytokines [14, 15, 16, 17], and that the expression of alarmin‐like cytokines (IL‐33, IL‐25, TSLP) may be upregulated in the epithelium during aspirin administration [4, 5, 8, 9]. However, in our study, neither the concentration of T2 cytokines nor alarmin‐like cytokines in ISS changed after aspirin ingestion.

We observed an increase in urinary LTE_4_ levels and a reduction in ISS levels of 15‐oxo‐ETE and LTB_4_ during aspirin‐induced bronchospasm compared with baseline. An increase in urinary LTE_4_ levels after aspirin ingestion in N‐ERD is well documented [43]. Reduced LTB_4_ levels in ISS after bronchial challenge with lysyl‐aspirin were noted in our previous research [39]. Recently, we also reported reduced ISS levels of 15‐oxo‐ETE in patients with N‐ERD following oral aspirin challenge [43].

We assessed the differences between patients with eosinophilic and noneosinophilic asthma phenotypes. No significant differences were found in clinical variables. Patients with the airway eosinophilic inflammatory phenotype had higher blood eosinophil count and percentage, along with a higher expression of sputum cell genes related to T2 and eosinophil signatures, which is consistent with previous research [44]. No significant differences were found in blood ILCs, which is noteworthy given that previous studies documented that blood ILC2 levels were significantly higher in patients with eosinophilic asthma compared with those with noneosinophilic asthma [45]. Notably, patients with the eosinophilic phenotype exhibited increased sputum ILC3 percentage than those with the noneosinophilic phenotype, while no differences were found in ILC1 and ILC2 percentages. Interestingly, ILC3s may be associated with both eosinophilic and noneosinophilic asthma phenotypes, indicating that additional studies are needed to clarify the role of ILC3s in asthma [46].

We also aimed to compare the effect of oral aspirin on blood and sputum ILCs between patients with eosinophilic and noneosinophilic asthma phenotypes. Both groups showed a significant increase in blood ILC1 count and percentage, while no significant changes were found in blood ILC2s and ILC3s, nor in sputum ILCs, during aspirin‐induced bronchospasm compared with baseline in either group. This indicates that the asthma phenotype had no influence on the effect of aspirin on blood and sputum ILCs. No changes were observed in blood and ISS cytokine levels during aspirin‐induced bronchospasm compared with baseline in either group. At baseline, patients with the eosinophilic asthma phenotype had significantly higher ISS levels of LTE_4_ and 15‐HETE, along with lower ISS levels of LTB_4_, 5‐HETE, and 12‐HETE compared with those with the noneosinophilic asthma phenotype. Additionally, patients with eosinophilic asthma exhibited significantly higher urinary levels of LTE_4_ at baseline compared with those with noneosinophilic asthma. Urinary LTE_4_ levels correlated positively with both blood and sputum eosinophils, as well as eosinophil‐related genes from sputum cells. However, no significant correlations were found between blood ILCs and lipid mediators in urine and ISS.

Our study has several limitations. First, the blood and sputum ILC count was very low [37]. Second, it is likely that sputum is not an ideal material for studying ILCs, and more invasive methods such as bronchial mucosa sampling, bronchial scraping, or BAL should be considered. Third, we did not perform functional studies of ILCs because of very limited cell counts. Finally, CRTH2 was used to identify ILC2s, which can be downregulated in the presence of PGD_2_ [47].

In summary, N‐ERD patients demonstrated an increase in blood ILC1s after aspirin ingestion, while no changes in blood ILC2s and ILC3s were observed. Sputum ILC percentages were very low at baseline and during aspirin‐induced bronchospasm. Patients with the eosinophilic asthma phenotype had a higher sputum ILC3 percentage compared with those with the noneosinophilic phenotype.

## 4 Data Limitations and Perspectives

The primary limitation of our study is the small sample size, which is attributable to the rarity of N‐ERD occurrence. Another limitation is the timing of biological sample collection, which occurred immediately following the onset of aspirin‐induced bronchospasm. A later time point for sample collection may have provided additional insights. The timing was constrained by the necessity to administer medications to alleviate the patient's symptoms, which could have influenced the number of ILC cells present in the blood and sputum. Our findings suggest that induced sputum is not an optimal specimen for studying ILCs, and more invasive techniques, such as bronchial mucosal biopsy, bronchial scraping, or bronchoalveolar lavage (BAL), should be considered. Functional studies of ILCs were not performed due to the limited number of cells available. An important limitation of the present study is the absence of data from a murine model, which could have provided additional insights into the dynamics of ILCs in the lower airways. While the use of an animal model would have strengthened our findings and allowed for further mechanistic exploration, it was beyond the scope and resources of the current study.

## 5 Materials and Methods

### 5.1 Characteristics of the Patients

The study included 24 patients with N‐ERD. All subjects had stable asthma without any exacerbations in the 6 weeks preceding hospital admission, with a forced expiratory volume in 1 s of 70% or higher on the day of admission. All participants were treated with inhaled corticosteroids and long‐acting β_2_‐agonists. None of the patients received systemic glucocorticoids or antileukotrienes during the 6 weeks prior to the study. Patients who had previously received biologic treatment were excluded from the study. The baseline characteristics of patients are presented in Table 2. The study was approved by the Bioethics Committee of Jagiellonian University (no. 1072.6120.234.2020; date of issue: October 24, 2019). All study participants gave written consent to participate in the study. Details are presented in the Supporting Information.

### 5.2 Study Design and Sample Collection

Patients were recruited during an outpatient visit 6 weeks prior to the main study. All patients underwent a 4‐day hospitalization for an oral aspirin challenge. During the first day of hospitalization, medical history, physical examination, questionnaire tests, spirometry, and sinus computed tomography were performed, and induced sputum, blood, and urine samples were collected. On the second and third days of hospitalization, all patients underwent a single‐blind placebo‐controlled oral aspirin challenge [49]. During aspirin‐induced bronchospasm, induced sputum, blood, and urine samples were collected again. Sputum induction was performed according to the European Respiratory Society guidelines [50] and following the same protocol as in our previous studies [50, 51]. Four phenotypes based on sputum cell percentages were distinguished [51]. Details are presented in the Supporting Information.

### 5.3 Flow Cytometry

Blood ILC1s were identified as CD45+, LIN−, CD127+, CD117−, and CRTH2− cells; ILC2s as CD45+, LIN−, CD127+, CD117−, and CRTH2+ cells; and ILC3s as CD45+, LIN−, CD127+, CD117+, and CRTH2− cells [21, 28]. Detection threshold for blood ILCs was ∼0.001% of viable CD45+ blood cells, which corresponded to approximately 1 cell per 1 µL of blood. Sputum ILC1s were identified as CD45+, LIN−, CD127+, CD117, and CRTH2− cells; ILC2s as CD45+, LIN−, CD127+, CD117−, and CRTH2+ cells; and ILC3s as CD45+, LIN−, CD127+, CD117+, and CRTH2− cells. Due to technical limitations related to the lower cell count in sputum specimens, a less restrictive detection threshold for ILCs in sputum samples was adopted, set at approximately ∼0.01% of viable CD45+ sputum cells. Details are provided in the Supporting Information. Gating strategy for blood ILCs is illustrated in Figure E1 in the Supporting Information. A list of antibodies is presented in Table E3 in the Supporting Information.

### 5.4 Lipid Mediators

The concentration of 17 eicosanoids including leukotrienes LTB_4_, LTC_4_, LTD_4_, and LTE_4_, prostaglandins PGD_2_ and PGE_2_, 12‐hydroxyeicosatetraenoic acid (12‐HETE), 15‐HETE, and 15‐oxo‐eicosatetraenoic acid (15‐oxo‐ETE) was measured in induced sputum supernatant (ISS) by high‐performance liquid chromatography‐tandem mass spectrometry (HPLC‐MS/MS) (AB Sciex, Washington, US, Triple Quat 5500+) [51]. The concentration of urinary eicosanoids, including LTE_4_, was measured by HPLC‐MS/MS (AB SCIEX, QTrap 4000) [51]. Details are provided in the Supporting Information.

### 5.5 Cytokines

Serum and ISS cytokine concentrations were determined using the commercial enzyme immunoassay method with R&D Systems kits on a Luminex 200 analyzer. ISS results were expressed as picograms per 1 mg of protein. The lower limits of detection are presented in the Supporting Information.

### 5.6 mRNA Expression

The relative mRNA expression of T2 and eosinophil‐related genes: *CCR3*, *CLC*, *CLCA1*, *CST1*, *POSTN*, *PRSS33*, and *SERPINB2* was assessed using TaqMan qPCR (ThermoFisher Scientific). Data were normalized to a housekeeping gene (*GAPDH*) [52].

### 5.7 Statistical Analysis

Statistical analyses were performed using TIBCO Software Inc. Statistica v.13, integrated with the R environment. The Benjamini–Hochberg procedure was used to control the false discovery rate for multiple comparisons. A *p*‐value of less than 0.05 was considered significant. Details are provided in the Supporting Information.

## Author Contributions

Lucyna Mastalerz conceived and designed the project. Radosław Kacorzyk and Alicja Maciejska acquired the data. Radosław Kacorzyk, Bogdan Jakiela, Adam Ćmiel, Marek Sanak, and Agnieszka S. Węgrzyn analyzed and interpreted the data. Radosław Kacorzyk and Lucyna Mastalerz wrote the paper.

## Ethics Approval Statement for Human Studies and Patient Consent Statement

The study was approved by the Bioethics Committee of Jagiellonian University (no. 1072.6120.234.2020; date of issue: 10/24/2019). All study participants gave written consent to participate in the study.

## Conflicts of Interest

The authors declare no conflicts of interest.

## Peer Review

The peer review history for this article is available at https://publons.com/publon/10.1002/eji.70020.

## Supporting information




**Supporting File 1**: eji70020‐sup‐0001‐SuppMat.pdf

## Data Availability

The data that support the findings of this study are available from the corresponding author upon reasonable request.
