# Supplementary material for: Effect of Aspirin Challenge on Innate Lymphoid Cells in Asthma Patients With Aspirin Hypersensitivity
Source: Eur J Immunol. 2025 Aug 4;55(8):e70020. doi: 10.1002/eji.70020 (PMC12319354; doi:10.1002/eji.70020)
Supplement: Supplementary file 1 — Supporting File 1: eji70020‐sup‐0001‐SuppMat.pdf [file EJI-55-e70020-s001.pdf]

Article title: **Effect of aspirin challenge on innate lymphoid cells in asthma patients with aspirin hypersensitivity**

Supporting Information

Methods

Characteristics of the patients

The study included 24 patients with N-ERD. All subjects had stable asthma without any exacerbations in the 6 weeks preceding hospital admission, with a forced expiratory volume in 1 second of 70% or higher on the day of admission. All participants were treated with inhaled corticosteroids and long-acting β_2-agonists. None of the patients received systemic glucocorticoids or antileukotrienes during the 6 weeks prior to the study. Patients who had previously received biologic treatment were excluded from the study. CRSwNP was confirmed in all patients by a laryngologist. Asthma control was assessed in accordance with the 2022 Global Initiative for Asthma guidelines[1] and using the standard questionnaires, including the Asthma Control Test (ACT) and the 7-item Asthma Control Questionnaire (ACQ-7). The severity of nasal symptoms was assessed using the Sino-Nasal Outcome Test-22 (SNOT-22) scale.[2] Sinus computed tomography scans were graded by two experienced radiologists using the Lund-Mackay score.[3] The baseline characteristics of patients are presented in Table 2 in Main Manuscript. The study was approved by the Bioethics Committee of Jagiellonian University (no. 1072.6120.234.2020; date of issue: 10/24/2019). All study participants gave written consent to participate in the study.

Study design and sample collection

Patients were recruited during an outpatient visit 6 weeks before the main study. After the 6-week run-in period, all subjects underwent a 4-day hospitalization, during which an oral aspirin challenge was performed. The study design is presented in Figure E2. During the first day of hospitalization, a history, physical examination, questionnaire tests, spirometry, and sinus computed tomography were performed, and induced sputum, blood, and urine samples were collected. On the second and third day of hospitalization, all patients underwent single-blind placebo-controlled oral aspirin challenge according to the EAACI/GA2LEN guidelines.[4] During aspirin-induced bronchospasm, induced sputum, blood, and urine samples were collected again. Blood samples were used to isolate peripheral blood mononuclear cells (PBMCs), which were cryopreserved. Serum aliquots were frozen at -80°C for subsequent cytokine evaluation. Urine supernatant aliquots were frozen at -80°C for further eicosanoid evaluation. Sputum induction was performed according to the European Respiratory Society guidelines[5] and following the same protocol as in our previous studies.[6-8] Sputum was processed to obtain cytospin preparations, and induced sputum supernatant (ISS) was frozen at -80°C for cytokine and eicosanoid evaluation. Centrifugated cell pellets were used for flow cytometry ILC measurements and mRNA isolation. Differential cell counts were performed on May-Grunwald-Giemsa staining and expressed as percentage of non-squamous cells.[6] Four phenotypes based on sputum cell percentages were distinguished: eosinophilic (≥3% eosinophils and <60% neutrophils), neutrophilic (≥60% neutrophils and <3% eosinophils), mixed (≥3%, eosinophils and ≥60% neutrophils), and paucigranulocytic (<3% eosinophils and <60% neutrophils).[6, 9]

Flow cytometry

Flow cytometry analysis was performed according to “Guidelines for the use of flow cytometry and cell sorting in immunological studies (third edition)”.[10] ILCs were measured in sputum samples and cryopreserved PBMCs. For the detection of circulating ILCs, PBMC samples were thawed, washed twice with Roswell Park Memorial Institute (RPMI) 1640 medium (all basic reagents from Sigma Aldrich, Saint Louis, U.S., if not otherwise specified) with addition of DNAse (0.1 mg/mL), and checked for viability (>95% in all samples after 1 h rest). Next, aliquots of $3x10^6$ of PBMCs were washed with phosphate-buffered saline (PBS) and stained in PBS with a viability dye (Fixable Viability Dye eFluor 780, Thermo Fisher Scientific, Walthman, U.S.) for 30 min at 4°C. After incubation samples were washed with staining buffer (PBS containing 0.5% BSA and 2 mM EDTA) and then labeled for 30 min at 4°C with the following monoclonal antibodies (all from BioLegend, San Diego, U.S.): a LIN set of FITC-conjugated antibodies, including CD1a (clone HI149), CD3 (OKT3), CD11c (3.9), CD14 (HCD14), CD16 (3G8), CD19 (HIB19), CD20 (2H7), CD34 (561), CD56 (HCD56), CD94 (DX22), CD123 (6H6), FcεRIα (AER-37), CD303 (201A), TCR α/β (IP26), and TCR γ/δ (B1). The antibodies used to identify ILCs included PerCP/Cy5.5-CD161 (HP-3G10), PE/Cy7-CD127 (anti-IL-7Rα; A019D5), BV421-CD117 (anti-c-kit; 104D2), BV510-CD45 (HI30), and AF647-CD294 (anti-CRTH2; BM16). The samples were then washed and acquired with a FACSCanto™ II flow cytometer (Becton Dickinson, Franklin Lakes, U.S.) with recording of at least 1.5 to $2 x10^6$ viable cell events. Detection threshold for blood ILCs was approximately 0.001% of viable CD45+ blood cells, which corresponded to approximately 1 cell per 1 µL of blood. ILC1 were identified as CD45+, LIN-, CD127+, CD117-, and CRTH2- cells; ILC2 as CD45+, LIN-, CD127+, CD117-, and CRTH2+ cells; and ILC3 as CD45+, LIN-, CD127+, CD117+, and CRTH2- cells.[11, 12] The gating strategy for

blood ILCs is presented in Figure E1 in the Supplementary Material. List of flow cytometry antibodies is presented in Table E3 in the Supplementary Material.

For the detection of ILCs in sputum, approximately two-thirds of processed sputum sample was centrifuged to obtain cell pellets, washed with phosphate buffer, and filtered with a 70-μm mesh. We then proceeded immediately with the flow cytometry procedure using the same staining protocol as used for PBMCs. Due to the variable quality of sputum and the often reduced number of cells in the sample, we recorded the maximum possible number of events during acquisition (usually between 100-600 $x10^3$). Therefore, the ILC detection threshold was higher compared to blood samples (~0.01% of viable CD45+ sputum cells). Similar to blood, sputum ILC1s were identified as CD45+, LIN-, CD127+, CD117, and CRTH2- cells; ILC2 as CD45+, LIN-, CD127+, CD117-, and CRTH2+ cells; and ILC3 as CD45+, LIN-, CD127+, CD117+, and CRTH2- cells.[11, 12]

Lipid mediators

The concentrations of ISS eicosanoids and their respective lower limits of detection (LLOD) were measured by high-performance liquid chromatography-tandem mass spectrometry (HPLC-MS/MS) (AB Sciex, Washington, US, Triple Quat 5500+): leukotriene B_4 (LTB_4, LLOD 0.346 pg/mg protein), leukotriene C_4 (LTC_4, 0.437), leukotriene D_4 (LTD_4, 0.339), leukotriene E_4 (LTE_4, 0.215), eoxin C_4 (EXC_4), eoxin D_4 (EXD_4), eoxin E_4 (EXE_4, 0.222), lipoxin A_4 (LXA_4), PGD_4 (0.251), prostaglandin E_4 (PGE_4, 0.353), tetranor-prostaglandin D metabolite (tetranor-PGDM), tetranor-prostaglandin E metabolite (tetranor-PGEM), 5-hydroxyeicosatetraenoic acid (5-HETE, 0.077), 5-oxo-eicosatetraenoic acid (5-oxo-ETE, 0.193), 12-hydroxyeicosatetraenoic acid (12-HETE, 0.062), tatranor-12-hydroxyeicosatetraenoic acid (tetranor-12-HETE), 15-HETE (0.209), 15-oxo-eicosatetraenoic acid (15-oxo-ETE).[13, 14] The results of ISS eicosanoids were recalculated in picograms per mg

of protein (pg/mg protein). The concentrations of urinary eicosanoids were measured by HPLC-MS/MS (AB SCIEX, QTrap 4000): LTE_4 (0.57 pg/mg creatinine), PGD_2 (0.67), PGE2 (0.94), 5-HETE (0.2), 5-oxo-ETE (0.19), 12-HETE (0.16), 15-HETE (0.55), and 15-oxo-ETE.[13, 14] The results of urine eicosanoids were recalculated in picograms per mg of creatinine (pg/mg creatinine).

Cytokines

Serum and ISS cytokine concentrations were determined using the commercial enzyme immunoassay method with R&D Systems kits on a Luminex 200 analyzer. Serum cytokines and their respective LLoDs were as follows: IL-4 (0.22 pg/mL), IL-5 (1.18), IL-6 (2.0), IL-8 (CXCL8) (0.55), IL-9 (49.7), IL-13 (9.3), IL-17 (0.44), IL-25 (4.0), IL-33 (2.58), and TSLP (0.3). ISS results were standardized to the protein concentration and were expressed as pg per 1 mg of protein. ISS cytokines and their respective LLoDs were as follows: IL-4 (0.081), IL-5 (0.433), IL-6 (0.734), IL-8 (CXCL8) (0.2), IL-9 (18.241), IL-13 (3.413), IL-17 (0.161), IL-25 (1.468), IL-33 (0.947), and TSLP (0.11).

Statistics

Statistical analyses were performed using TIBCO Software Inc. Statistica v.13 integrated with the R environment. Summary statistics for demographic, clinical, and laboratory characteristics were presented as medians with 25th and 75th percentiles for continuous variables, and as the number and percentage of total for categorical data. The Wilcoxon test was used for comparisons of dependent samples before and during the positive oral aspirin challenge. The Mann-Whitney U test was used for comparisons of independent samples. Spearman rank correlation coefficients were used as a measure of monotonic dependence.

The Benjamini-Hochberg procedure was used to control the false discovery rate in multiple comparisons. A p-value of less than 0.05 was considered statistically significant.

Figure E1. Gating strategy for blood innate lymphoid cells (ILCs). ILC1s were identified as CD45+, LIN-, CD127+, CD117-, and CRTH2- cells; ILC2s as CD45+, LIN-, CD127+, CD117-, CRTH2+ cells; and ILC3s as CD45+, LIN-, CD127+, CD117+, and CRTH2- cells. PBMC, peripheral blood mononuclear cell.

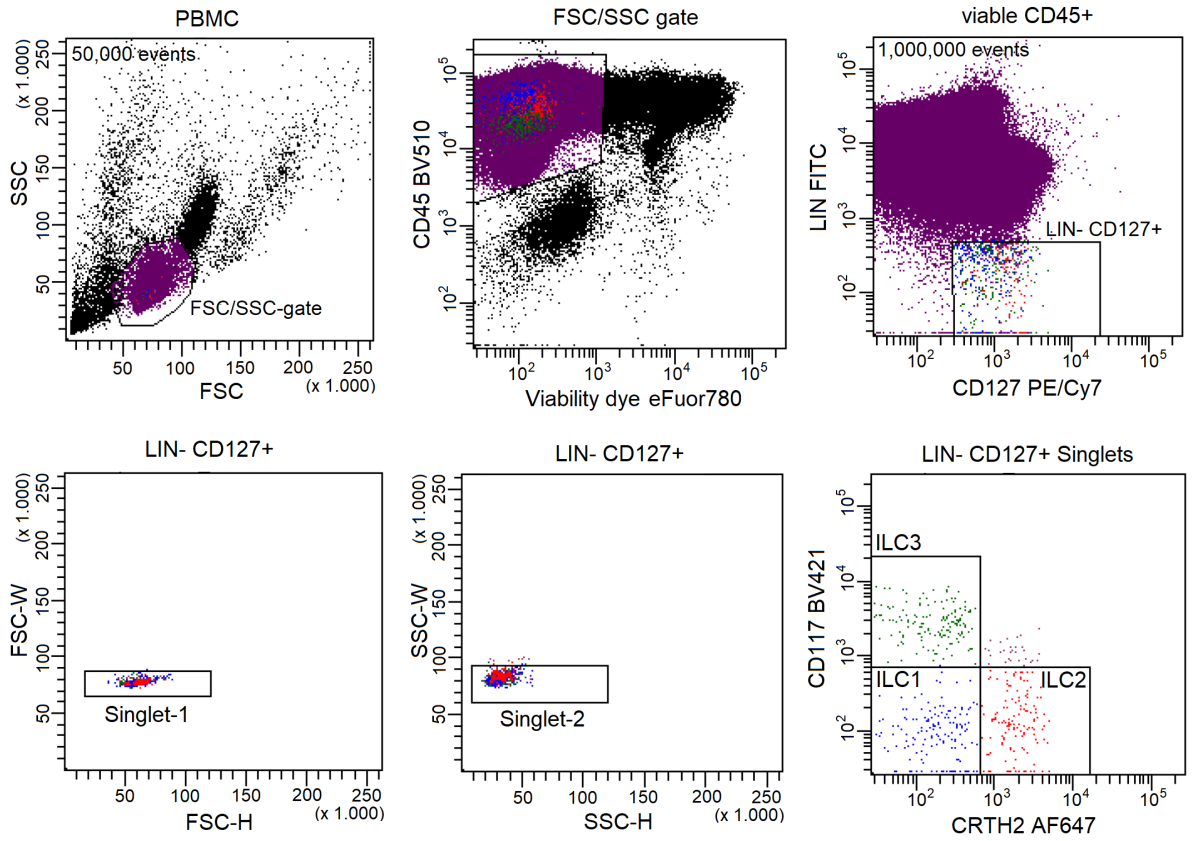

Figure E2. Study design and sample collection. Sputum, blood, and urine samples were collected from all study patients (n=24) twice: at baseline and during aspirin-induced bronchospasm. Abbreviations: ILC1, group 1 innate lymphoid cells; ILC2, group 2 innate lymphoid cells; ILC3, group 3 innate lymphoid cells.

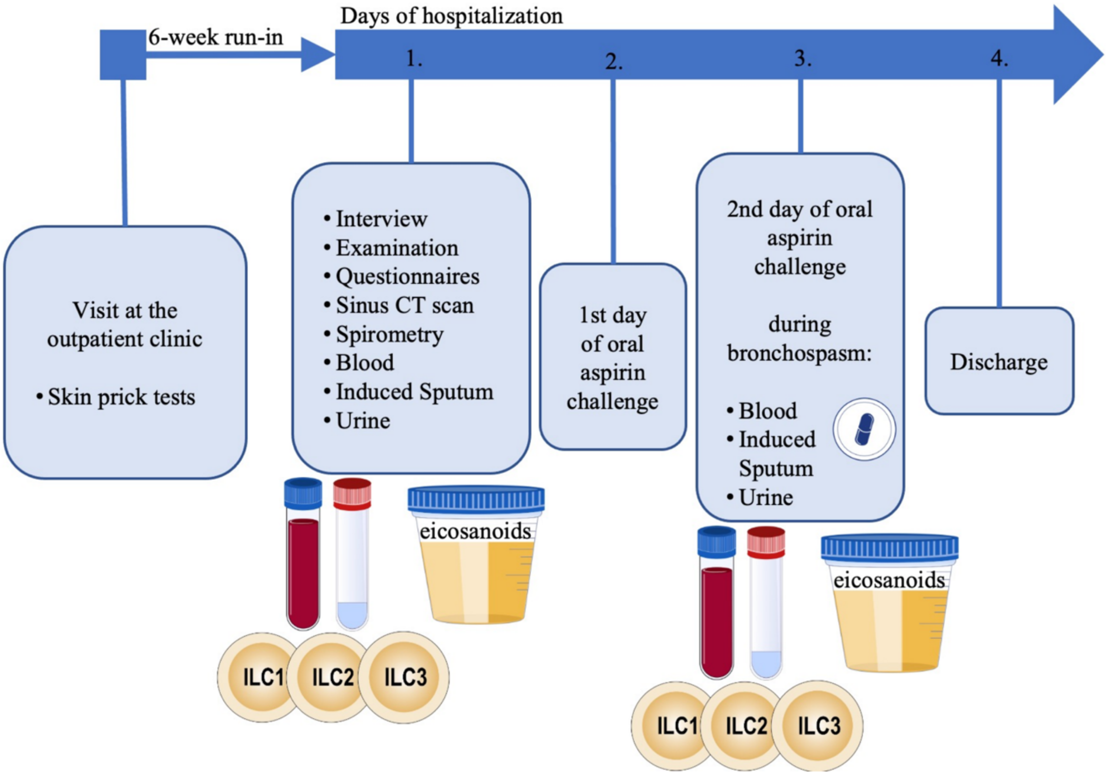

Table E1. Sputum and urine parameters at baseline and during aspirin-induced bronchospasm in the N-ERD cohort (n=24). Supplement to Table 1 in the Main Manuscript.

Variable	At baseline	During aspirin-induced bronchospasm	BH adjusted p-value
Induced sputum cells [% of non-squamous cells]			
Neutrophils	32.01 [17.51; 45.27]	34.66 [21.03; 46.61]	0.209
Eosinophils	1.24 [0; 9.60]	0.76 [0; 8.65]	0.647
Lymphocytes	1.01 [0.77; 1.30]	0.71 [0.59; 1.13]	0.110
Monocytes	0.59 [0.19; 1.15]	0.31 [0; 0.61]	0.094
Macrophages	55.95 [41.01; 78.13]	55.04 [39.59; 72.70]	0.253
Asthma phenotypes based on sputum cell percentage			
Eosinophilic	10 (42%)	9 (38%)	1.0
Neutrophilic	1 (4%)	1 (4%)	
Paucigranulocytic	13 (54%)	13 (54%)	
Mixed	0 (0%)	1 (4%)	
Sputum transcriptome [GAPDH relative expression log10)			
CCR3	1.97 [1.62; 2.54]	2.19 [1.61; 2.47]	0.820
CLC	1.36 [1.14; 2.33]	1.51 [1.03; 2.39]	0.976
CLCA1	-0.16 [-1.0; 0.71]	0.07 [-1.0; 1.65]	0.248
CST1	1.07 [0.29; 1.48]	1.64 [0.71; 2.17]	0.083
POSTN	-1.0 [-1.0; -0.50]	-1.0 [-1.0; 0.34]	0.742
PRSS33	0.86 [0.27; 1.67]	0.93 [-0.21; 1.68]	0.663
SERPINB2	2.38 [2.15; 2.74]	2.42 [1.77; 2.75]	0.808
ISS cytokine concentration [pg/mg protein]			
Interleukin-4	0.09 [0.08; 0.15]	0.08 [0.08; 0.11]	0.838
Interleukin-5	0.43 [0.43; 0.44]	0.43 [0.43; 0.59]	0.292
Interleukin-6	15.18 [7.66; 53.41]	7.08 [4.05; 25.08]	0.243
Interleukin-8 (CXCL8)	569.61 [299.64; 1125.24]	311.40 [184.31; 478.09]	0.243
Interleukin-9	18.24 [18.24; 25.28]	18.24 [18.24; 29.4]	0.838
Interleukin-13	7.05 [4.85; 9.64]	5.46 [3.41; 9.07]	0.823
Interleukin-17	0.75 [0.4; 1.21]	0.72 [0.23; 1.07]	0.357
Interleukin-25	1.47 [1.47; 1.51]	1.47 [1.47; 1.47]	0.866
Interleukin-33	3.32 [1.1; 6.56]	3.0 [1.13; 4.14]	0.511
TSLP	0.27 [0.11; 0.5]	0.29 [0.15; 0.7]	0.838
ISS eicosanoid concentration [pg/mg protein]			
Tetranor-PGDM	4.86 [3.19; 6.33]	5.98 [3.13; 9.15]	0.258
Tetranor-PGEM	8.12 [6.70; 11.40]	9.26 [7.01; 14.23]	0.337
5-oxo-ETE	113.98 [44.94; 180.71]	42.42 [29.35; 154.54]	0.189
Eoxin C4	8.29 [4.81; 18.12]	7.22 [3.90; 15.13]	0.617
Eoxin D4	15.20 [9.06; 33.05]	9.95 [5.01; 18.15]	0.097
Eoxin E4	1.65 [0.99; 4.02]	1.40 [0.77; 3.12]	0.395
Lipoxin A4	10.47 [6.32; 19.03]	6.94 [3.54; 14.38]	0.292
Urinary eicosanoids [pg/mg creatinine]			

Tetranor-PGDM	1429.09 [1035.25; 1760.43]	1652.37 [1158.56; 1932.78]	0.230
Tetranor-PGEM	5134.96 [2662.66; 6019.36]	2792.09 [1473.25; 5824.17]	0.486
5-HETE	4.77 [3.73; 9.13]	6.44 [4.63; 16.87]	0.278
5-oxo-ETE	0.27 [0.19; 0.82]	0.56 [0.35; 2.70]	0.486
15-HETE	12.05 [7.7; 20.85]	15.84 [9.45; 24.61]	0.277
15-oxo-ETE	6.54 [5.05; 11.56]	9.65 [5.17; 16.88]	0.633

Values are expressed as medians [0.25; 0.75 quartiles] or n (%).
A p-value of less than 0.05 was considered statistically significant.
Abbreviations: BH – Benjamini-Hochberg; ISS – induced sputum supernatant; TSLP – thymic stromal lymphopoietin; 5-HETE – 5-hydroxyeicosatetraenoic acid; 5-oxo-ETE – 5-oxo-eicosatetraenoic acid; 15-HETE – 15-hydroxyeicosatetraenoic acid; 15-oxo-ETE – 15-oxo-eicosatetraenoic acid

Table E2. Differences in sputum and urine eicosanoids between N-ERD patients with eosinophilic (n=10) and noneosinophilic asthma phenotype (n=14) at baseline. Supplement to Table 3 in the Main Manuscript.

Variable	Patients with eosinophilic asthma phenotype (n=10)	Patients with non-eosinophilic asthma phenotype (n=14)	BH-adjusted p-value
ISS eicosanoid concentration [pg/mg protein]			
Tetranor-PGDM	4.65 [2.65; 5.70]	5.5 [3.78; 6.57]	0.403
Tetranor-PGEM	7.49 [6.70; 11.63]	9.39 [6.66; 11.32]	0.708
5-oxo-ETE	60.92 [32.25; 131.46]	137.94 [89.67; 184.46]	0.084
Eoxin C_4	6.90 [2.6; 12.37]	8.77 [4.84; 21.5]	0.585
Eoxin D_4	15.83 [10.98; 25.22]	15.20 [8.63; 35.17]	0.752
Eoxin E_4	2.64 [1.19; 4.57]	1.16 [0.31; 3.99]	0.235
Lipoxin A_4	14.14 [4.9; 19.79]	10.10 [6.61; 18.78]	0.886
Urinary eicosanoids [pg/mg creatinine]			
Tetranor-PGDM	1659.29 [1014.59; 2270.74]	1309.11 [1055.92; 1750.23]	0.371
Tetranor-PGEM	5375.87 [2794.42; 7881.24]	4684.63 [1841.26; 5666.43]	0.352
5-HETE	5.68 [3.74; 11.11]	4.73 [3.73; 7.22]	0.508
5-oxo-ETE	0.23 [0.19; 0.81]	0.29 [0.19; 0.94]	1.0
15-HETE	15.07 [8.18; 19.14]	11.35 [7.22; 21.33]	0.885
15-oxo-ETE	5.11 [4.55; 14.13]	6.9 [6.07; 9.45]	0.432

Values are expressed as medians [with 0.25; 0.75 quartile] or n (%).
A p-value of less than 0.05 was considered statistically significant.
Abbreviations: BH – Benjamini-Hochberg; ISS – induced sputum supernatant; 5-HETE – 5-hydroxyeicosatetraenoic acid; 5-oxo-ETE – 5-oxo-eicosatetraenoic acid; 15-HETE – 15-hydroxyeicosatetraenoic acid; 15-oxo-ETE – 15-oxo-eicosatetraenoic acid

Table E3. List of flow cytometry antibodies.

Antibody	Clone	Fluorochrome	Cat#
anti-CD1a	HI149	FITC	300104
anti-CD3	OKT3	FITC	317306
anti-CD11c	3.9	FITC	301604
anti-CD14	HCD14	FITC	325604
anti-CD16	3G8	FITC	302006
anti-CD19	HIB19	FITC	302206
anti-CD20	2H7	FITC	302304
anti-CD34	561	FITC	343604
anti-CD56 (NCAM)	HCD56	FITC	318304
anti-CD94	DX22	FITC	305504
anti-CD123	6H6	FITC	306014
anti-FcεRIα	AER-37	FITC	334608
anti-CD303 (BDCA-2)	201A	FITC	354208
anti-TCR α/β	IP26	FITC	306706
anti-TCR γ/δ	B1	FITC	331208
anti-CD45	HI30	Brilliant Violet 510	304036
anti-CD294 (CRTH2)	BM16	Alexa Fluor 647	350104
anti-CD127 (IL-7Rα)	A019D5	PE/Cy7	351320
anti-CD117 (c-kit)	104D2	Brilliant Violet 421	313216

All reagents were purchased from BioLegend (San Diego, U.S.).

FITC labeled antibodies were used as a lineage (LIN) mix.

References:

1. 2022 Gina Main Raport https://ginasthma.org/wp-content/uploads/2023/05/GINA-Main-Report-2022-WMSA.pdf
2. Haque R, White AA, Jackson DJ, Hopkins C. Clinical evaluation and diagnosis of aspirin-exacerbated respiratory disease. *J Allergy Clin Immunol*. 2021;148(2):283-291. doi:10.1016/j.jaci.2021.06.018
3. Lund VJ, Kennedy DW. Staging for rhinosinusitis. *Otolaryngol Head Neck Surg*. 1997;117(3 Pt 2):S35-S40. doi:10.1016/S0194-59989770005-6
4. Nizankowska-Mogilnicka E, Bochenek G, Mastalerz L, et al. EAACI/GA2LEN guideline: aspirin provocation tests for diagnosis of aspirin hypersensitivity. *Allergy*. 2007;62(10):1111-1118. doi:10.1111/j.1398-9995.2007.01409.x
5. Djukanović R, Sterk PJ, Fahy JV, Hargreave FE. Standardised methodology of sputum induction and processing. *Eur Respir J Suppl*. 2002;37:1s-2s. doi:10.1183/09031936.02.00000102
6. Tyrak KE, Kupryś-Lipińska I, Czarnobilska E, et al. Sputum biomarkers during aspirin desensitization in nonsteroidal anti-inflammatory drugs exacerbated respiratory disease. *Respir Med*. 2019;152:51-59. doi:10.1016/j.rmed.2019.04.021
7. Tyrak KE, Pajdzik K, Konduracka E, et al. Artificial neural network identifies nonsteroidal anti-inflammatory drugs exacerbated respiratory disease (N-ERD) cohort. *Allergy*. 2020;75(7):1649-1658. doi:10.1111/all.14214
8. Mastalerz L, Kacorzyk R, Jakieła B, Ćmiel A, Sanak M. Sputum transcriptome analysis of co-regulated genes related to arachidonic acid metabolism in N-ERD. *Allergy*. 2023;78(2):553-555. doi:10.1111/all.15501

9. Simpson JL, Scott R, Boyle MJ, Gibson PG. Inflammatory subtypes in asthma: assessment and identification using induced sputum. *Respirology*. 2006;11(1):54-61. doi:10.1111/j.1440-1843.2006.00784.x
10. Cossarizza, A., Chang, H. D., Radbruch, A., Abrignani, S., Addo, R., Akdis, M., Andrä, I., et al., Guidelines for the use of flow cytometry and cell sorting in immunological studies (third edition) Eur. J. Immunol. 2021. 51: 2708–3145
11. Golebski K, Ros XR, Nagasawa M, et al. IL-1β, IL-23, and TGF-β drive plasticity of human ILC2s towards IL-17-producing ILCs in nasal inflammation. *Nat Commun*. 2019;10(1):2162. Published 2019 May 14. doi:10.1038/s41467-019-09883-7
12. Eastman JJ, Cavagnero KJ, Deconde AS, et al. Group 2 innate lymphoid cells are recruited to the nasal mucosa in patients with aspirin-exacerbated respiratory disease. *J Allergy Clin Immunol*. 2017;140(1):101-108.e3. doi:10.1016/j.jaci.2016.11.023
13. Tyrak KE, Kupryś-Lipińska I, Czarnobilska E, et al. Sputum biomarkers during aspirin desensitization in nonsteroidal anti-inflammatory drugs exacerbated respiratory disease. *Respir Med*. 2019;152:51-59. doi:10.1016/j.rmed.2019.04.021
14. Tyrak KE, Pajdzik K, Konduracka E, et al. Artificial neural network identifies nonsteroidal anti-inflammatory drugs exacerbated respiratory disease (N-ERD) cohort. *Allergy*. 2020;75(7):1649-1658. doi:10.1111/all.14214
